# A phase 3 randomized placebo-controlled trial of darbepoetin alfa in patients with anemia and lower-risk myelodysplastic syndromes

**DOI:** 10.1038/leu.2017.192

**Published:** 2017-07-14

**Authors:** U Platzbecker, A Symeonidis, E N Oliva, J S Goede, M Delforge, J Mayer, B Slama, S Badre, E Gasal, B Mehta, J Franklin

**Affiliations:** 1 University Hospital Carl Gustav Carus Dresden, Medizinische Klinik und Poliklinik I, Dresden, Germany; 2 Division of Hematology, Department of Internal Medicine, University of Patras Medical School, Patras, Greece; 3 Division of Hematology, Azienda Ospedaliera Bianchi-Melacrino-Morelli, Reggio Calabria, Italy; 4 Division of Hematology, University Hospital and University of Zürich, Zürich, Switzerland; 5 Department of Hematology & Chairman Leuven Cancer Institute, University Hospital Leuven, Leuven, Belgium; 6 Department of Internal Medicine-Hematology and Oncology, University Hospital Brno and Faculty of Medicine, Masaryk University, Brno, Czech Republic; 7 Oncologie Médicale-Hématologie Clinique, Centre Hospitalier Departemental, Avignon, France; 8 Amgen Inc., Thousand Oaks, CA, USA

## Abstract

The use of darbepoetin alfa to treat anemia in patients with lower-risk myelodysplastic syndromes (MDS) was evaluated in a phase 3 trial. Eligible patients had low/intermediate-1 risk MDS, hemoglobin ⩽10 g/dl, low transfusion burden and serum erythropoietin (EPO) ⩽500 mU/ml. Patients were randomized 2:1 to receive 24 weeks of subcutaneous darbepoetin alfa 500 μg or placebo every 3 weeks (Q3W), followed by 48 weeks of open-label darbepoetin alfa. A total of 147 patients were randomized, with median hemoglobin of 9.3 (Q1:8.8, Q3:9.7) g/dl and median baseline serum EPO of 69 (Q1:36, Q3:158) mU/ml. Transfusion incidence from weeks 5–24 was significantly lower with darbepoetin alfa versus placebo (36.1% (35/97) versus 59.2% (29/49), *P*=0.008) and erythroid response rates increased significantly with darbepoetin alfa (14.7% (11/75 evaluable) versus 0% (0/35 evaluable), *P*=0.016). In the 48-week open-label period, dose frequency increased from Q3W to Q2W in 81% (102/126) of patients; this was associated with a higher hematologic improvement–erythroid response rate (34.7% (34/98)). Safety results were consistent with a previous darbepoetin alfa phase 2 MDS trial. In conclusion, 24 weeks of darbepoetin alfa Q3W significantly reduced transfusions and increased rates of erythroid response with no new safety signals in lower-risk MDS (registered as EudraCT#2009-016522-14 and NCT#01362140).

## Introduction

Patients with low or intermediate-1 (int-1) International Prognostic Scoring System (IPSS) risk myelodysplastic syndromes (MDS) have a median survival of 5.7 and 3.5 years, respectively, when treated with supportive care, that is, primarily red blood cell (RBC) transfusion support.^1^ The main concern for these patients is anemia, a hallmark of MDS which mostly defines the disease burden in low/int-1 risk MDS.^2^ Anemia symptoms have various manifestations and alter patients’ quality of life. Accordingly, many patients receive RBC transfusions, which can lead to iron overload, cardiac and liver morbidity and mortality, and are associated with worse prognosis.^2^ Prevention or reduction of transfusions is therefore the main goal of clinical care in lower-risk MDS.

Erythropoiesis-stimulating agents (ESAs), such as epoetin alfa or darbepoetin alfa, are recommended in clinical guidelines to treat anemia in patients with lower-risk MDS, who generally have low transfusion burden.^3, 4, 5^ These recommendations are based on several phase 2 and 3 trials,^6, 7, 8, 9, 10, 11, 12, 13, 14, 15, 16, 17, 18, 19, 20, 21, 22, 23, 24^ including a phase 3 trial of daily erythropoietin (EPO) with or without granulocyte colony-stimulating factor with supportive care versus supportive care alone.^8^ In this trial there was an increased erythroid response rate with EPO treatment per International Working Group (IWG) 2000 criteria;^25^ response to EPO was associated with improved survival.

This is in line with other reports that patients responding to ESAs have a more favorable prognosis, with lower probability of progressing to acute myeloid leukemia (AML) and longer overall survival.^8, 17, 26, 27^ Main predictors of response are transfusion burden and endogenous EPO levels.^28^ In a recent meta-analysis of 55 trials, transfusion independence was associated with decreased mortality (hazard ratio of 0.41; 95% confidence interval (CI): 0.29–0.56).^29^ However, improved survival with ESAs has not been demonstrated in prospective randomized trials. Despite all the clinical trials, ESAs are not approved globally for use in MDS (approved in the European Union, Japan and Turkey). This lack of approval, mainly due to the paucity of placebo-controlled data, could limit access and impact patient care.

Here we describe the results of the largest prospective, phase 3, randomized, placebo-controlled multicenter trial to date of ESA treatment in lower-risk MDS, evaluating efficacy and safety of subcutaneous darbepoetin alfa in patients with IPSS low/int-1 risk MDS, anemia and low transfusion burden.

## Patients and methods

### Patients and study design

**Figure 1:**
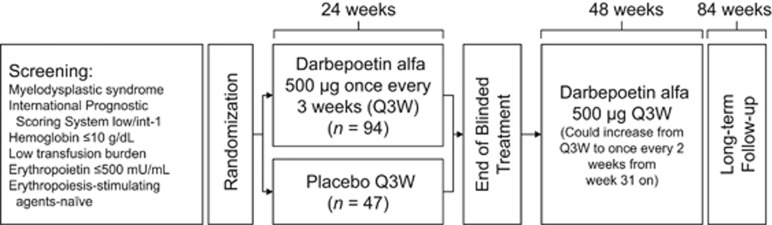
Study design.

This was a phase 3, randomized, double-blind, placebo-controlled study of darbepoetin alfa in ESA-naive patients with low/int-1 risk MDS and anemia (Figure 1, https://eudract.ema.europa.eu/index.html, EudraCT#2009-016522-14 and https://clinicaltrials.gov/, NCT#01362140). The study was approved by the relevant institutional review boards/ethics committees and conducted in accordance with the Helsinki Declaration. Each patient signed an approved informed consent form.

Eligible patients, enrolled in nine European countries from December 2011 to August 2014, had MDS per World Health Organization 2008 criteria with IPSS low/int-1 risk (determined locally), anemia (hemoglobin ⩽10 g/dl), endogenous serum EPO ⩽500 mU/ml, low transfusion burden (<4 RBC transfusion units in each of two consecutive 8-week periods before randomization), no previous treatment with ESAs or disease-modifying treatments and no bone marrow fibrosis. Patients were stratified by screening IPSS risk (low/int-1) for 2:1 randomization to 24 weeks of darbepoetin alfa or placebo followed by 48 weeks of open-label darbepoetin alfa for all patients and ongoing long-term follow-up for up to 3 years on study (although there is no investigational product (IP) during follow-up, any treatment, including commercially available ESAs, was allowed, per investigator). Transfusion endpoints were assessed from week 5 onward to allow adequate time for the effects of darbepoetin alfa to be observed.

### Endpoints

Based on a phase 2 trial,^6^ an estimated 40% hematologic improvement–erythroid response (HI-E) per IWG 2006 criteria was used to calculate the sample size, with HI-E defined as ⩾1.5 g/dl increase from baseline in hemoglobin with a mean rise of ⩾1.5 g/dl for 8 weeks.^30^ During routine monitoring and review of blinded data by Amgen in 2013 (with ~1/2 expected trial patients), the cumulative HI-E response rate was lower than expected, making it less likely to detect a difference. Therefore, before unblinding and while enrollment was ongoing, Amgen, with input from regulatory bodies, recommended that transfusion incidence from weeks 5–24 become the primary endpoint and HI-E a secondary endpoint. This switch of endpoints was accepted by regulatory bodies in all countries except for Germany, where the Federal Institute for Drugs and Medical Devices (Bundesinstitut für Arzneimittel und Medizinprodukte) proceeded with the original endpoints (HI-E as primary, transfusion incidence as secondary).

### Randomization and blinding

Eligible patients were randomized to darbepoetin alfa or placebo in a 2:1 ratio via an interactive voice response system, based on a schedule generated by Amgen before study start. Randomization was stratified by screening IPSS risk (low versus int-1). During the double-blind treatment period, the patient, site personnel and Amgen staff were blinded to treatment assignments. Darbepoetin alfa was provided as a clear, colorless, sterile, preservative-free, polysorbate solution in single-dose vials containing 100, 200, 300 or 500 μg/ml of darbepoetin alfa. Placebo was provided in identical containers.

### Dosing

During the 24-week double-blind treatment period, patients received darbepoetin alfa 500 μg or matched placebo subcutaneously once every 3 weeks (Q3W) from day 1/week 1 to week 22. During the active treatment period (week 25 on), all patients could receive darbepoetin alfa 500 μg Q3W, except those who had dose reductions, who continued with the last dose assigned. Dose escalation was permitted only from weeks 31–71. For patients with a hemoglobin increase of <1.5 g/dl (relative to week 1 for patients randomized to darbepoetin alfa and week 25 for patients randomized to placebo) in the absence of RBC transfusions in the previous 28 days, the dose was escalated from 500 μg Q3W to 500 μg once every 2 weeks (Q2W) for the remainder of the active treatment period. Once switched to Q2W dosing, patients did not switch back to Q3W dosing (even if the dose was later reduced) to minimize visit schedule changes.

If a patient’s hemoglobin reached >12 g/dl, the dose was withheld until hemoglobin decreased to ⩽11.0 g/dl; then treatment was restarted at a reduced dose. If hemoglobin increased by >1.5 g/dl in 21 days for Q3W dosing or >1.0 g/dl in 14 days for Q2W dosing, in the absence of RBC transfusions, the dose was reduced. IP was discontinued and the patient entered long-term follow-up if >3 dose reductions were needed. Patients received whole blood or packed RBC transfusions when hemoglobin decreased to ⩽8.0 g/dl or as clinically indicated (for example, symptomatic).

### Assessments

At each visit in the 24-week blinded and 48-week active treatment periods, patients were assessed for adverse events and thrombovascular events, and complete blood count and hemoglobin values were obtained. Ongoing independent central review was performed when local assessments found disease progression to AML or myelofibrosis or reticulin fibrosis. At weeks 1, 13, 25, 31, 42/43, 54/55 and 72/73, quality-of-life (QoL) assessments, Functional Assessment of Chronic Illness Therapy-Fatigue and EuroQol five dimensions visual analog scale were performed. Antibodies against darbepoetin alfa and EPO were assessed at weeks 1 and 25.

For exploratory independent expert review, blinded data were provided for hemoglobin (central and local), IP dose and schedule, any discontinuations or withdrawals for safety and transfusions. The three external experts reviewed each patient for clinical erythroid response, which was based on IWG 2006 criteria and also included changes in response duration if IP was discontinued for hemoglobin >12 g/dl, response to Q2W dosing during the active-treatment period, clinically meaningful transfusion threshold changes during the study (versus screening) and that baseline hemoglobin elevated by recent transfusions would affect response assessment.

### Statistical analysis

The primary endpoint, RBC transfusion incidence weeks 5–24, was analyzed using a *χ*^2^-test for differences in proportions, with sensitivity analyses using the Cochran-Mantel–Haenszel *χ*^2^-test stratified by IPSS risk. For HI-E rates, a Cochran-Mantel–Haenszel *χ*^2^-test stratified by IPSS risk was used, with a sensitivity analysis using the *χ*^2^-test.

Endpoints were hierarchically tested to maintain the overall significance of 0.05. Hypothesis testing used a two-sided significance of 0.05. For all other endpoints, statistical testing was descriptive and no further adjustments were made for multiplicity. The planned sample size was set to 141; assumptions included 85% power, two-sided *α* of 5%, dropout rate of 5% and 135 evaluable patients (software: nQuery Advisor version 7.0).

## Results

### Patient characteristics and disposition

**Figure 2:**
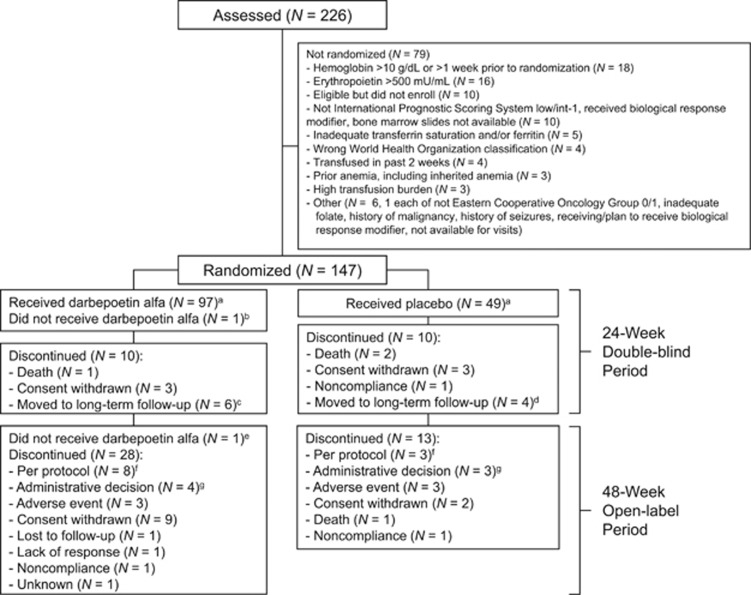
Patient disposition. ^a^Primary analysis sets. ^b^Serum EPO⩽500 mU/ml locally but >500 mU/ml centrally, so patient was withdrawn. ^c^For adverse event (*n*=2), protocol (*n*=2), administrative (*n*=1) and other (*n*=1). ^d^For adverse event (*n*=2), consent withdrawn (*n*=1) and protocol (*n*=1). ^e^Colon adenocarcinoma (T1aN0M0) treated with polypectomy. ^f^Per dosing algorithm (*n*=9), progression to AML (*n*=2, both prior darbepoetin alfa). ^g^Per investigator (*n*=2), lack of response (*n*=2) and unknown (*n*=3).

Patients (*N*=147) were randomized 2:1 to receive darbepoetin alfa (*n*=98) or placebo (*n*=49); baseline demographic and disease characteristics were generally similar (Table 1). Double-blind period completion rates were darbepoetin alfa: 89% (87/98); placebo: 80% (39/49). At the end of the 48-week open-label period, 117 patients continued in long-term follow-up (Figure 2).

### Efficacy: transfusions, HI-E and QoL

**Figure 3:**
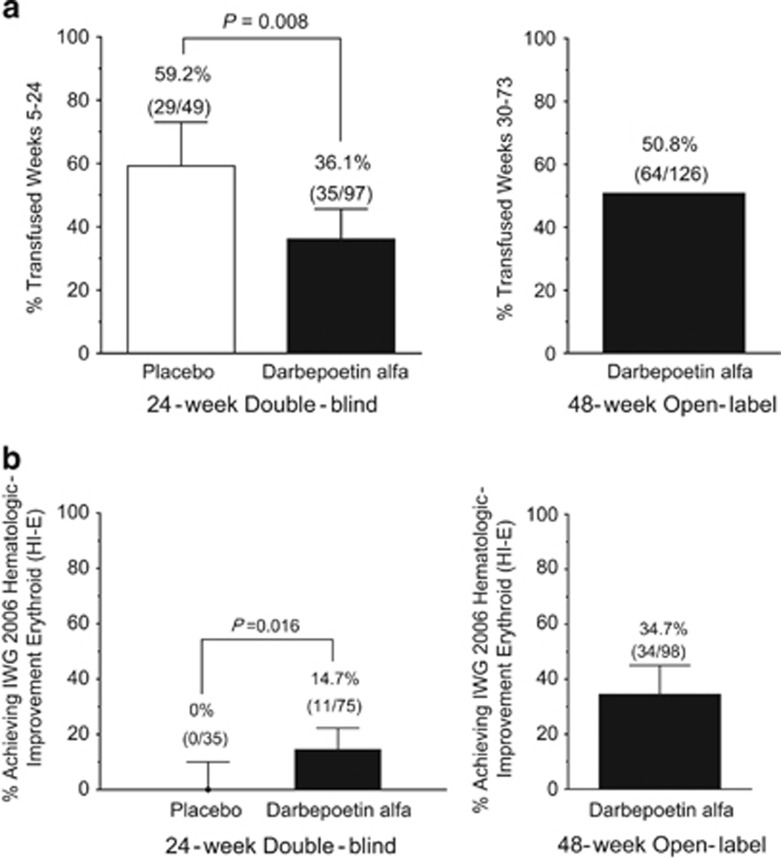
Efficacy of darbepoetin alfa. (**a**) Transfusions assessed from week 5 onward to allow for the effects of darbepoetin alfa to be observed. (**b**) Erythroid response rates; reasons unevaluable for HI-E in the double-blind period (*n*=37) included transfusion in prior 28 days (*n*=33), no hemoglobin measurement within 14 days of first dose (*n*=3), did not receive darbepoetin alfa (*n*=1).

Transfusion incidence from weeks 5–24 was significantly reduced with darbepoetin alfa (odds ratio (95% CI) 0.38 (0.19–0.79), darbepoetin alfa: 36.1% (35/97), placebo: 59.2% (29/49), *P*=0.008) (Figure 3a). The mean (s.e.) numbers of transfusion episodes were darbepoetin alfa: 1.4 (0.2) and placebo: 2.7 (0.5) (*P*<0.001), and units transfused were darbepoetin alfa: 2.2 (0.4) and placebo: 4.1 (0.9) (*P*=0.038). Transfusion rates were less with lower baseline EPO for darbepoetin alfa (⩽100 mU/ml: 23%, >100 mU/ml: 57%, 95% CI non-overlapping) but not placebo. The proportion achieving HI-E per IWG 2006 criteria was significantly increased with darbepoetin alfa versus placebo in the 24-week double-blind period (darbepoetin alfa: 14.7% (95% CI: 7.6–24.7%) (11/75 evaluable), placebo: 0% (95% CI: 0-10%) (0/35 evaluable), *P*=0.016) (Figure 3b, unevaluable reasons in figure legend, response rate and disposition in Supplementary Figure S1). An erythroid response rate in favor of darbepoetin alfa was also found by *post hoc* exploratory independent blinded expert panel review (darbepoetin alfa: 23.6% (21/89), placebo: 4.2% (2/48)). All patients with HI-E (*n*=11) in the double-blind period had baseline serum EPO ⩽100 mU/ml, 4/11 had a dose withheld for hemoglobin >12 g/dl and 10/11 had no transfusions in the 16 weeks before randomization (none had transfusions in the 8 weeks before). For the 24-week period, the HI-E rate for each World Health Organization category was refractory anemia: 22% (2/9), refractory anemia with ringed sideroblasts: 12% (2/17), refractory cytopenias with multilineage dysplasia: 13% (6/45) and refractory anemia with excess blasts-1: 8% (1/13) (0% for other categories). A comparison of responses per IWG 2006 and historical IWG 2000 criteria is in Supplementary Table S1. When transfusion incidence and HI-E were examined by IPSS and revised IPSS (IPSS-R) risk (Supplementary Figure S2 and Supplementary Table S1), whereas changes by IPSS were not notably different, decreased transfusion requirements were associated with more favorable IPSS-R (*P*=0.005 for IPSS-R related to transfusion rates, *P*=0.56 for IPSS-R related to HI-E; both in the entire patient population).

In the 48-week open-label period, the transfusion rate was 50.8% (64/126) and the HI-E rate was 34.7% (34/98) overall, 28.9% (11/38) for prior placebo and 26.4% (23/87) for prior darbepoetin alfa (10 of the prior darbepoetin alfa had HI-E in the 24-week portion). Of the 34 patients with HI-E, 6/34 (18%) had transfusions in the 16 weeks before randomization, 30/34 (88%) received Q2W dosing at some point, 30/34 (88%) had baseline serum EPO⩽100 mU/ml and 26/34 (76%) had doses withheld. For the 48-week period, the HI-E rate for each World Health Organization category was refractory anemia: 35% (7/20), refractory anemia with ringed sideroblasts: 15% (3/20), refractory cytopenias with multilineage dysplasia: 25% (13/53), del 5q: 23% (3/13), refractory anemia with excess blasts-1: 41% (7/17), MDS-U: 50% (1/2) and unknown: 0% (0/1). The mean (s.e.) duration of response was 235 (21) days, including both the 24-week double-blind and 48-week open-label treatment periods; 21/34 patients with HI-E were still responding at last observation.

For QoL assessments, for the 24-week double-blind period, the mean (s.d.) change from baseline with the EuroQol five dimensions visual analog scale did not differ by treatment (darbepoetin alfa (*n*=81): 2.1 (13.1) points, placebo (*n*=39): 0.8 (15.7) points). No difference was seen in rates of clinically meaningful (⩾3-point) improvement in Functional Assessment of Chronic Illness Therapy-Fatigue subscale score (darbepoetin alfa (*n*=85): 35.6% (95% CI: 25.7–46.4%), placebo (*n*=39): 31.0% (95% CI: 17.6–47.1%)). Further analyses of QoL data for the 24-week double-blind period by HI-E status, IPSS (low versus int-1), transfusion history (present/absent), hemoglobin response and stratification by median baseline QoL did not yield any significant differences. Likewise, for the subsequent 48-week open-label period, similar results were seen for prior darbepoetin alfa and prior placebo for EuroQol five dimensions visual analog scale and Functional Assessment of Chronic Illness Therapy-Fatigue.

### Exposure

In the 24-week double-blind period, dose was reduced due to a rapid rise in hemoglobin (>1.5 g/dl in 3 weeks) in 18 (18%) darbepoetin alfa patients at a median (min–max) hemoglobin of 10.6 (8.4–12) g/dl (Table 2). Eleven (11%) darbepoetin alfa patients had a dose withheld for hemoglobin >12 g/dl; 4 of these 11 met HI-E criteria. In comparison, no placebo patients had the IP dose reduced or withheld.

In the 48-week open-label period, the median average dose administered was 500 μg. Fifty patients (40%) had a dose reduced a total of 85 times at a median (min–max) hemoglobin of 10.6 (8.5–11.9) g/dl. Dose was withheld for hemoglobin >12 g/dl in 36 (29%) patients. Dose frequency was increased from Q3W to Q2W in 81% (102/126) of patients.

### Safety

During the double-blind treatment period, most adverse events were grade 1–2 in severity (Table 3). Adverse events leading to IP discontinuation were reported in two placebo-treated patients (grade 3 pulmonary arterial hypertension, grade 3 renal failure) and three darbepoetin alfa-treated patients (grade 3 pulmonary thrombosis, grade 3 thrombocytopenia, grade 1 increased blast cell count). There was no particular pattern for cardiovascular adverse events, thrombovascular adverse events, or adverse events of grade ⩾4 severity (Supplementary Tables S2, S3, and S4 respectively).

In the double-blind period, serious adverse events were reported in 11 darbepoetin alfa-treated patients (11.2%) and eight placebo-treated patients (16.7%) (Supplementary Table S5). The three fatal adverse events were hemorrhagic proctitis in the darbepoetin alfa group and one case each of cardiac failure and cerebral hemorrhage in the placebo group. The most frequently reported adverse events were patient-reported fatigue (darbepoetin alfa: 17.3%, placebo: 8.3%), asthenia (darbepoetin alfa: 12.2%, placebo: 10.4%) and exertional dyspnea (darbepoetin alfa: 6.1%, placebo: 10.4%) (Supplementary Table S6).

The incidence of disease progression to AML was similar in the darbepoetin alfa and placebo groups (2.1% versus 2.2%) (Supplementary Table S7). All AML cases were confirmed by central pathology; per central review, two patients who developed AML were refractory anemia with excess blasts-2 at baseline, not refractory anemia with excess blasts-1 as determined locally. Per protocol, these patients discontinued IP after AML diagnosis. One darbepoetin alfa-treated patient was diagnosed with stage 1A colon adenocarcinoma (T1aN0M0) 4 months after initiating treatment and was treated with polypectomy. No neutralizing antibodies to darbepoetin alfa or EPO were detected in those with post-baseline results (darbepoetin alfa *N*=91, placebo *N*=43). Regarding neutrophils and platelets, no significant differences from baseline or between groups were observed.

Adverse events reported in the 48-week open-label period were generally similar to those observed in the 24-week treatment period and similar between prior placebo and prior darbepoetin alfa groups (Table 3 and Supplementary Tables S2–S7). IP discontinuation due to adverse events occurred in three prior darbepoetin alfa patients (lung disorder, tetany, MDS progression, renal disorder and deep vein thrombosis) and three prior placebo patients (pulmonary embolism, anemia and delirium). Deaths included one of the AML cases (prior darbepoetin alfa) and pneumonitis (prior placebo). There were no neutralizing antibodies found to either darbepoetin alfa or EPO in this period (number of patients with post-baseline results: prior darbepoetin alfa *N*=80, prior placebo *N*=35).

## Discussion

In this first phase 3, randomized, double-blind, placebo-controlled prospective trial of subcutaneous darbepoetin alfa in patients with IPSS low/int-1 risk MDS and anemia, darbepoetin alfa Q3W for 24 weeks significantly reduced transfusion incidence and increased rates of erythroid response per IWG 2006 criteria. These results are particularly notable as, in daily practice, the aim in managing patients with lower-risk MDS is to achieve transfusion independence, which is associated with improved survival.^8, 17, 26, 27, 29^ Safety findings were consistent with the known darbepoetin alfa safety profile and the phase 2 trial,^6^ with no new safety signals identified and an AML incidence of 2% in each group during the double-blind treatment period. Of note, we did not observe a significant increase in thromboembolic adverse events, which is consistent with previously reported results.^24^ The safety profile in the 48-week open-label period was similar for patients who had previously received placebo and those previously receiving darbepoetin alfa, indicating that longer darbepoetin alfa exposure was not associated with increased risk of adverse events. Although no significant changes were seen in QoL, the study was not powered for these analyses and the relatively small number of responders made it difficult to detect improved QoL. Further, that all patients, independent of treatment, received transfusions as needed could have made it harder to detect a difference in QoL.

Data from the 48-week open-label darbepoetin alfa period show an increased HI-E rate compared with the 24-week double-blind period; this may reflect that 81% of patients increased their dose frequency from Q3W to Q2W in the 48-week open-label period (increased dose frequency only allowed weeks 31–71). This HI-E rate is comparable to that of a phase 3 placebo-controlled study of epoetin-alfa in patients with low/int-1 MDS (31.8% versus 4.4% at 24 weeks); dosing in the epoetin-alfa study could be escalated after 8 weeks and adjusted weekly thereafter. The increased response rate may also reflect that prolonged treatment may be needed to obtain the full clinical benefit.^31^ These data indicate that a substantial number of patients receiving the 500 μg Q3W regimen may have been underdosed (as described by Fenaux and Adès^32^), accounting for the low response rate, and that Q2W dosing may offer more clinical benefit. In support of that, a recent systematic review of 10 darbepoetin alfa MDS studies found greater response rates in patients receiving doses equivalent to those in the open-label period of this trial.^16^ Further, the substantial number of patients (42%) with a history of RBC transfusions (albeit still low transfusion burden) may have influenced the outcomes of this trial.

The nature of the stringent IWG 2006 HI-E criteria (1.5 g/dl hemoglobin increase maintained for 8 weeks, even in patients with low transfusion burden) likely led to a lower than expected clinical benefit rate. The independent expert panel, which in addition to IWG 2006 criteria considered several variables before and during the study on a patient-by-patient basis, identified more patients with a clinical erythroid response as compared with the assessment strictly per the published version of the IWG 2006 criteria without additional clinical considerations. These data highlight important clinical factors not incorporated or reflected by current IWG criteria; these factors should be considered for any future revisions of IWG criteria.

Detecting erythroid response was further complicated by the trial design in several ways. First, hemoglobin was measured every 3 weeks, so in practice, patients had to maintain response over 9 weeks. Second, the darbepoetin alfa dose was held if hemoglobin rose to >12 g/dl and decreased if hemoglobin increased by >1.5 g/dl in 3 weeks (or 1 g/dl in 2 weeks for those dosed Q2W). This resulted in some patients having to reduce the darbepoetin alfa dose while still anemic, with dose reductions occurring with hemoglobin as low as 8.4 g/dl. Early dose reduction in patients with a rapid hemoglobin rise who were still anemic could have hampered the achievement of sustained hemoglobin responses and thus could have lowered the erythroid response rate. Third, as mentioned earlier, during the 48-week open-label period, 81% of patients changed from Q3W to Q2W dosing, indicating that optimal dosing was not achieved during the 24-week double-blind period. When erythroid response was measured as meeting IWG 2000 criteria at any point (that is, not necessarily for 8 weeks), higher response rates were seen (major response: 19%, minor response: 39% Supplementary Table S1).

Our findings are in keeping with other ESA MDS trials, indicating that our results are likely generalizable to other patients with lower-risk MDS and anemia. Previously, in a phase 3 trial of EPO with supportive care versus supportive care alone, an increased erythroid response rate was observed with EPO, with no difference observed in survival or transformation to AML, with a median follow-up of 5.8 years.^8^ Retrospective analyses of Spanish and Greek MDS registries of patients with lower-risk MDS found that many patients responded to ESAs.^27, 33^ Further, ESA treatment in MDS was associated with improved survival and no increase in AML in a cohort study.^26, 27^ We also confirm the predictive value of IPSS-R risk with regard to transfusion response; thus, IPSS-R risk, in addition to baseline transfusion burden and endogenous EPO levels, can serve as an important marker to guide clinical care.

In conclusion, in this large phase 3, randomized, double-blind, placebo-controlled trial of darbepoetin alfa in IPSS low/int-1 risk MDS patients with anemia, subcutaneous darbepoetin alfa significantly reduced transfusions and increased rates of erythroid response compared with placebo with no new safety signals. Thus, darbepoetin alfa, at a dose of 500 μg given every 2 weeks, can be a meaningful treatment option for low/int-1 risk MDS patients suffering from anemia.

## Figures and Tables

**Table 1:** Patient characteristics

		*Placebo (*n=*49)*	*Darbepoetin alfa (*n=*97)*	*Total (*n=*146)*
Male		29 (59.2)	51 (52.6)	80 (54.8)
Ethnicity, Caucasian		49 (100.0)	97 (100.0)	146 (100.0)
Age, years, median (Q1, Q3)		73 (66, 80)	74 (68, 79)	74 (67, 79)
				
*IPSS risk category*^a^				
Low		25 (51.0)	49 (50.5)	74 (50.7)
Intermediate-1		24 (49.0)	48 (49.5)	72 (49.3)
				
*IPSS-R*^b^ *risk category*^a^				
Very low		5 (10.2)	9 (9.3)	14 (10.0)
Low		27 (55.1)	62 (63.9)	89 (60.9)
Intermediate		13 (26.5)	20 (20.6)	33 (22.6)
High		1 (2.0)	3 (3.1)	4 (2.7)
				
*WHO 2008 classifications*^a^				
RA		13 (26.5)	9 (9.3)	22 (15.1)
RARS		4 (8.2)	17 (17.5)	21 (14.4)
RCMD		19 (38.8)	45 (46.4)	64 (43.8)
MDS-U		1 (2.0)	1 (1.0)	2 (1.4)
Del 5q		2 (4.1)	11 (11.3)	13 (8.9)
RAEB-1^c^		10 (20.4)	13 (13.4)	23 (15.8)
Unknown		0	1 (1.0)	1 (0.7)
				
*IPSS karyotype*^a^				
Good		44 (89.8)	89 (91.8)	133 (91.1)
Intermediate		5 (10.2)	8 (8.2)	13 (8.9)
Poor		0	0	0
				
*Bone marrow blasts*				
<5%		40 (81.6)	83 (85.6)	123 (84.2)
5–10%		9 (18.4)	14 (14.4)	23 (15.8)
⩾11%		0	0	0
				
*Cytopenias*^d^				
0–1		28 (57.1)	60 (61.9)	88 (60.3)
2–3		21 (42.9)	37 (38.1)	58 (39.7)
Time since MDS Dx, months, median (Q1, Q3)		4.3 (2.7, 12.6)	4.3 (2.0, 15.1)	4.3 (2.2, 14.5)
Hemoglobin, g/dl, median (Q1, Q3)		9.3 (8.8, 9.5)	9.3 (8.7, 9.8)	9.3 (8.8, 9.7)
Endogenous serum EPO level, mU/ml, median (Q1, Q3)		73.5 (35.8, 168)	66.1 (38, 150)	68.6 (35.9, 158)
RBC transfusions in 16 weeks before Randomization^e^	0 units	26 (53.1)	59 (60.8)	85 (58.2)
	1–3 units	11 (22.4)	25 (25.8)	36 (24.7)
	⩾4 units	12 (24.5)	13 (13.4)	25 (17.1)

Abbreviations: Del 5q, myelodysplastic syndrome associated with isolated 5q deletion; Dx, diagnosis; EPO, erythropoietin; IPSS, International Prognostic Scoring System; IPSS-R, revised IPSS; MDS, myelodysplastic syndrome; MDS-U, myelodysplastic syndrome, unclassified; Q1, Q3, quartile 1, quartile 3; RA, refractory anemia; RAEB-1, refractory anemia with excess blasts-1; RARS, refractory anemia with ringed sideroblasts; RBC, red blood cell; RCMD, refractory cytopenias with multilineage dysplasia; WHO, World Health Organization.

Data are *n* (%) unless indicated otherwise. One patient randomized to darbepoetin alfa did not receive any investigational product and is not included here.

a IPSS, WHO and cytogenetic classifications were determined locally; IPSS-R classifications were determined centrally, but based on local data.

b Not all patients had data available to classify by IPSS-R, so percentages will not add up to 100.

c One placebo patient with 3% marrow blasts also had 2% blood myeloblasts, and so was classified as RAEB-1. Another placebo patient with 1% marrow blasts was categorized as RAEB-1 per investigator. A darbepoetin alfa patient with 1% marrow blasts had 6% blasts on prior assessments and so was categorized as RAEB-1 per investigator. Another darbepoetin alfa patient with 1% marrow blasts was categorized as RAEB-1 per local pathologist. A third darbepoetin alfa patient with 3% marrow blasts had erythroblasts accounting for >50% of the cellularity and thus, per investigator, was categorized as RAEB-1 based on non-erythroid count.

d Cytopenias were defined as hemoglobin <10 g/dl, absolute neutrophil count <1.5 × 10^9^/l or platelets <100 × 10^9^/l.

e When transfusions were assessed in the 8 weeks before randomization, two patients in each group were high-transfusion (⩾4 units). Inclusion of these patients was a protocol violation.

**Table 2:** Exposure

	*24-Week double-blind period*		*48-Week open-label DAR*	
	*Placebo (*n=*48)*	*DAR (*n=*98)*	*Prior placebo (*n=*38)*	*Prior DAR (*n=*87)*
Received all eight doses	37 (77)	77 (79)	NA	NA
Number of doses received	NA	8 (1–8)	20 (3–23)	19 (1–23)
Average dose (μg)	NA	500 (25–500)	500 (300–500)	500 (193–500)
				
*Dose reduced due to rapid Hgb rise*				
Once	0	16 (16)	7 (18)	18 (21)
Twice	0	2 (2)	5 (13)	11 (13)
3 Times	0	0	5 (13)	3 (3)
4 Times	0	0	1 (3)	0
In patients who also had HI-E	0	4 (4)	9 (24)	18 (21)
Hgb for dose reduction, g/dl	NA	10.6 (8.4–12)	10.6 (8.5–11.9)	10.6 (8.5–11.9)
				
*Dose not administered*				
Withheld as Hgb >12 g/dl	0	11 (11)^a^	12 (32)	24 (28)
Doses withheld in patients who also had HI-E	0	4 (4)	8 (21)	18 (21)
Adverse event	5 (10)	2 (2)	1 (3)	7 (8)
Noncompliance	0	1 (1)	6 (16)	12 (14)
Other	1 (2)^b^	2 (2)^c^	4 (11)^d^	12 (14)^e^

Abbreviations: DAR, darbepoetin alfa; Hgb, hemoglobin; HI-E, hematologic improvement-erythroid response; IP, investigational product; NA, not applicable.

Data are median (min–max) or *n* (%).

a Dose was withheld once for six patients, twice for four patients, and three times for one patient.

b No IP on site.

c Reasons for other were investigator decision and no IP on site.

d Other included unknown (*n*=3) and no IP on site (*n*=1).

e Other included unknown (*n*=7) and one each of investigator decision, patient unable to go to clinic, interactive voice response system malfunction, investigator concern regarding high Hgb value and investigator felt there was a lack of efficacy.

**Table 3:** Overall safety

	*24-Week double-blind period*		*48-Week open-label DAR*	
	*Placebo (*n=*48)*	*DAR (*n=*98)*	*Prior placebo (*n=*38)*	*Prior DAR (*n=*87)*
AEs leading to IP discontinuation	2 (4.2)	3 (3.1)	3 (7.9)	3 (3.4)
Grade⩾3	13 (27.1)	15 (15.3)	9 (23.7)	27 (31.0)
Grade⩾4	6 (12.5)	5 (5.1)	4 (10.5)	9 (10.3)
Fatal AEs (none treatment-related)	2 (4.2)	1 (1.0)	1 (2.6)	1 (1.1)
Serious AEs	8 (16.7)	11 (11.2)	7 (18.4)	22 (25.3)
Treatment-related serious AEs	-	1 (1.0)	1 (2.6)	1 (1.1)
Thrombovascular events	-	1 (1.0)	3 (7.9)	3 (3.4)
Progression to AML	1 (2.2)	2 (2.1)	-	2 (2.3)

Abbreviations: AE, adverse event; AML, acute myeloid leukemia; DAR, darbepoetin alfa; IP, investigational product.

Data are *n* (%). One patient randomized to placebo received a dose of DAR and so is included in that group. One patient enrolled into the 48-week open-label portion but did not receive any DAR; thus, total *N*=125 (not 126).
