# Supplementary material for: A phase 3 randomized placebo-controlled trial of darbepoetin alfa in patients with anemia and lower-risk myelodysplastic syndromes
Source: Leukemia. 2017 Jul 14;31(9):1944–50. doi: 10.1038/leu.2017.192 (PMC5596208; doi:10.1038/leu.2017.192)
Supplement: Supplementary Tables and Figures [file leu2017192x1.docx]

# SUPPLEMENTAL TABLES/FIGURES

**Supplemental tables**

| **Table S1.** Efficacy endpoints for 24-week blinded period by IPSS and IPSS-R risk | | | | | | | | | |
| --- | --- | --- | --- | --- | --- | --- | --- | --- | --- |
|  | | *Transfusions*  *Weeks 5-24* | | *HI-E per IWG 2006* | | *Major response per IWG 2000* | | *Minor response per IWG 2000* | |
|  |  | *PBO*  *(*n*=49)* | *DAR*  *(*n*=97)* | *PBO*  *(n=49)* | *DAR*  *(*n*=97)* | *PBO*  *(*n*=49)* | *DAR*  *(*n*=97)* | *PBO*  *(*n*=49)* | *DAR*  *(*n*=97)* |
| All patients | | 59 (29/49) | 36 (35/97) | 0 (0/35) | 15 (11/75) | 3 (1/35) | 19 (14/75) | 6 (2/35) | 39 (29/75) |
| IPSS | Low | 48 (12/25) | 33 (16/49) | 0 (0/18) | 18 (7/38) | 0 (0/18) | 24 (9/38) | 0 (0/18) | 39 (15/38) |
|  | Int-1 | 71 (17/24) | 40 (19/48) | 0 (0/17) | 11 (4/37) | 6 (1/17) | 14 (5/37) | 12 (2/17) | 38 (14/37) |
| IPSS-R | Very low | 60 (3/5) | 11 (1/9) | 0 (0/4) | 33 (2/6) | 25 (1/4) | 50 (3/6) | 25 (1/4) | 33 (2/6) |
|  | Low | 56 (15/27) | 27 (17/62) | 0 (0/22) | 16 (8/51) | 0 (0/22) | 18 (9/51) | 5 (1/22) | 37 (19/51) |
|  | Inter | 69 (9/13) | 65 (13/20) | 0 (0/7) | 7 (1/14) | 0 (0/7) | 14 (2/14) | 0 (0/7) | 43 (6/14) |
|  | High | 100 (1/1) | 67 (2/3) | 0 (0/1) | 0 (0/2) | 0 (0/1) | 0 (0/2) | 0 (0/1) | 50 (1/2) |
| Data are percent (x/*N*). HI-E is defined as ≥1.5 g/dL increase from baseline in hemoglobin with a mean rise of ≥1.5 g/dL for 8 weeks without transfusions as per IWG 2006 criteria.[^29^](#_ENREF_29) Per IWG 2000 criteria, a major erythroid response is defined as ≥2.0 g/dL increase from baseline in hemoglobin without transfusions in the past 4 weeks and a minor response is defined as ≥1.0 and <2.0 g/dL increase from baseline in hemoglobin without transfusions in the past 4 weeks[^33^](#_ENREF_33); here we describe response rates for meeting these criteria at 1 point in time (not necessarily for 8 weeks).  Abbreviations: DAR, darbepoetin alfa; HI-E, hematologic improvement-erythroid response; Int-1, intermediate 1; Inter, intermediate; IPSS, International Prognostic Scoring System; IPSS-R, revised IPSS; IWG, International Working Group; PBO, placebo. | | | | | | | | | |

| **Table S2.** Cardiovascular adverse events for the 24-week double-blind period | | | | | | | | |
| --- | --- | --- | --- | --- | --- | --- | --- | --- |
|  | *24-week double-blind period* | | | | *48-week open-label DAR* | | | |
|  | *Placebo*  *(*n*=48)* | | *DAR*  *(*n*=98)* | | *Prior placebo*  *(*n*=38)* | | *Prior DAR*  *(*n*=87)* | |
|  | n | *%* | n | *%* | n | *%* | n | *%* |
| Cardiac failure | 5 | 10.4 | 4 | 4.1 | 2 | 5.3 | 8 | 9.2 |
| Hypertension | 2 | 4.2 | 1 | 1.0 | 1 | 2.6 | - | - |
| Embolic and thrombotic events | - | - | 1 | 1.0 | 3 | 7.9 | 3 | 3.4 |
| Venous thromboembolic events | - | - | 1 | 1.0 | 2 | 5.3 | 1 | 1.1 |
| Central nervous system vascular disorders | 1 | 2.1 | - | - | 2 | 5.3 | 2 | 2.3 |
| Ischemic heart disease | 1 | 2.1 | - | - | - | - | 1 | 1.1 |
| One patient randomized to placebo received a dose of DAR and so is included in that group.  - indicates none.  Abbreviations: DAR, darbepoetin alfa. | | | | | | | | |

| **Table S3.** Thrombovascular adverse events | | | | | | | |
| --- | --- | --- | --- | --- | --- | --- | --- |
| *24-week double-blind period* | | | | *48-week open-label DAR* | | | |
| *Placebo*  *(*n*=48)* | | *DAR*  *(*n*=98)* | | *Prior placebo*  *(*n*=38)* | | *Prior DAR*  *(*n*=87)* | |
| n | *%* | n | *%* | n | *%* | n | *%* |
| - | - | 1 | 1.0 | 3 | 6.3 | 3 | 3.4 |
| None | | 52 year old white male with 2 PEs and 2 pulmonary thromboses over 1 month 7 weeks after starting DAR | | 82 year old white male, with CVA (0.9 years after starting DAR) and 2 PEs (both at 1 year) | | 70 year old white female with 2 TIAs 1.2 years after starting DAR | |
|  |  |  |  | 78 year old white female with 2 DVTs (both at 1.3 years) | | 71 year old white male with 2 DVTs (both 1.3 years after starting DAR) and 2 vascular stent occlusions (both 0.6 years after starting DAR) | |
|  |  |  |  | 79 year old white female with 2 TIAs (both at 0.8 years) | | 81 year old white female with PAD starting 0.8 years after starting DAR (ongoing) | |
| One patient randomized to placebo received a dose of DAR and so is included in that group. Abbreviations: CVA, cerebrovascular accident; DAR, darbepoetin alfa; DVT, deep venous thrombosis; PAD, peripheral arterial disease; PE, pulmonary embolism; TIA, transient ischemic attack. | | | | | | | |

| **Table S4.** Grade ≥4 adverse events | | | | | | | |
| --- | --- | --- | --- | --- | --- | --- | --- |
| *24-week double-blind period* | | | | *48-week open-label DAR* | | | |
| *Placebo*  *(*n*=48)* | | *DAR*  *(*n*=98)* | | *Prior placebo*  *(*n*=38)* | | *Prior DAR*  *(*n*=87)* | |
| n | *%* | n | *%* | n | *%* | n | *%* |
| 6 | 12.5 | 5 | 5.1 | 4 | 10.5 | 9 | 10.3 |
| Cerebral hemorrhage^a^ | | Anemia  Thrombocytopenia | | Pulmonary embolism | | Anemia  Thrombocytopenia | |
| Cardiac failure^a^ | | Pneumonia | | Hyperuricemia | | Carotid artery stenosis | |
| Thrombocytopenia | | Pulmonary embolism, chest pain | | Chronic cholecystitis | | Hip arthroplasty | |
| Hyperuricemia | | Staphylococcal pneumonitis and sepsis  Left foot necrosis  Acute renal failure  Hemorrhagic proctitis^a^ | | Pneumonitis^a^ | | Lower respiratory infection | |
| Neutropenia | | Neutropenia | |  | | Upper respiratory infection | |
| Asthenia | |  | |  | | Lower respiratory infection  Neutropenia | |
|  | |  | |  | | Neutropenia | |
|  | |  | |  | | Neutropenia | |
|  | |  | |  | | AML^a^ | |
| Abbreviations: AML, acute myeloid leukemia; DAR, darbepoetin alfa.  ^a^Patients who died due to adverse events. | | | | | | | |

| **Table S5.** Serious adverse events | | | | |
| --- | --- | --- | --- | --- |
|  | *24-week double-blind period* | | *48-week open-label DAR* | |
|  | *Placebo*  *(*n*=48)* | *DAR*  *(*n*=98)* | *Prior placebo*  *(*n*=38)* | *Prior DAR*  *(*n*=87)* |
| Anemia | - | 3 (3.1) | - | 2 (2.3) |
| Pneumonia^a^ | 2 (4.2) | 2 (2.0) | 1 (2.6) | 1 (1.1) |
| Renal failure | 2 (4.2) | - | - | - |
| Atrial fibrillation | - | - | - | 2 (2.3) |
| Lower RTI^a^ | - | - | - | 2 (2.3) |
| Abscess of salivary gland | - | 1 (1.0) | - | - |
| Acute kidney injury | - | 1 (1.0) | - | - |
| Asthenia | 1 (2.1) | - | - | - |
| Cardiac failure | 1 (2.1) | - | 1 (2.6) | - |
| Cerebral hemorrhage | 1 (2.1) | - | - | - |
| Cholecystectomy | - | 1 (1.0) | - | - |
| Gastrointestinal infection | 1 (2.1) | - | - | - |
| Hemorrhagic anemia | 1 (2.1) | - | - | - |
| Hemorrhagic proctitis | - | 1 (1.0) | - | - |
| Hypertension | 1 (2.1) | - | - | - |
| Leukocytosis | 1 (2.1) | - | - | - |
| Menorrhagia | - | 1 (1.0) | - | - |
| Necrosis in extremity | - | 1 (1.0) | - | - |
| Noncardiac chest pain | - | 1 (1.0) | - | - |
| Osteoarthritis | - | 1 (1.0) | - | - |
| Pneumonitis | - | 1 (1.0) | 1 (2.6) | - |
| Pulmonary arterial HTN | 1 (2.1) | - | - | - |
| Pulmonary embolism | - | 1 (1.0) | 1 (2.6) | - |
| Staphylococcal sepsis | - | 1 (1.0) |  |  |
| Thrombocytopenia/platelet count decreased^b^ | - | 1 (1.0) | - | 2 (2.2) |
| Urinary tract infection | - | 1 (1.0) | - | - |
| Urosepsis | 1 (2.1) | - | - | - |
| Weight decreased | 1 (2.1) | - | - | - |
| Vertigo | - | 1 (1.0) | - | 1 (1.1) |
| Abnormal liver function test | - | - | - | 1 (1.1) |
| Bronchitis | - | - | 1 (2.6) | 1 (1.1) |
| Calculus bladder | - | - | - | 1 (1.1) |
| Carotid artery stenosis | - | - | - | 1 (1.1) |
| Cataract | - | - | - | 1 (1.1) |
| Chronic cholecystitis | - | - | 1 (2.6) | - |
| Chronic obstructive pulmonary disease | - | - | - | 1 (1.1) |
| Circulatory collapse | - | - | - | 1 (1.1) |
| Delirium | - | - | 1 (2.6) | - |
| Device malfunction | - | - | - | 1 (1.1) |
| Dyspnea | - | - | - | 1 (1.1) |
| Enterococcal infection | - | - | - | 1 (1.1) |
| Erysipelas | - | - | - | 1 (1.1) |
| Fall | - | - | - | 1 (1.1) |
| Femur fracture | - | - | - | 1 (1.1) |
| Hip arthroplasty | - | - | - | 1 (1.1) |
| Hypersensitivity | - | - | - | 1 (1.1) |
| Nausea | - | - | - | 1 (1.1) |
| Neutropenia, febrile^c^ | - | - | - | 1 (1.1) |
| Neutropenia^c^ | - | - | - | 1 (1.1) |
| Palpitations | - | - | - | 1 (1.1) |
| Peripheral edema | - | - | - | 1 (1.1) |
| Pyrexia | - | - | - | 1 (1.1) |
| Rectal hemorrhage | - | - | 1 (2.6) | - |
| Sinoatrial block | - | - | - | 1 (1.1) |
| Stent placement | - | - | - | 1 (1.1) |
| Syncope | - | - | 1 (2.6) | - |
| Tetany | - | - | - | 1 (1.1) |
| Thoracic vertebral fracture | - | - | 1 (2.6) | - |
| Transient ischemic attack | - | - | 1 (2.6) | 1 (1.1) |
| Upper RTI | - | - | - | 1 (1.1) |
| Vascular stent occlusion^d^ | - | - | - | 1 (1.1) |
| Vascular stent thrombosis^d^ | - | - | - | 1 (1.1) |
| Vestibular disorder | - | - | - | 1 (1.1) |
| Data are n (%). Abbreviations: DAR, darbepoetin alfa; HTN, hypertension; RTI, respiratory tract infection.  ^a^The two patients with lower respiratory infections did not also have pneumonia. ^b^Three different patients; for the patient with “Platelet count decreased,” the platelet count fell to 3x10^9^/L. ^c^Two different patients. ^d^Same patient; thrombosis occurred 10 days after occlusion. | | | | |

| **Table S6.** Most frequent adverse events | | | | | | | | |
| --- | --- | --- | --- | --- | --- | --- | --- | --- |
|  | *24-week double-blind period* | | | | *48-week open-label DAR* | | | |
|  | *Placebo*  *(*n*=48)* | | *DAR*  *(*n*=98)* | | *Prior placebo*  *(*n*=38)* | | *Prior DAR*  *(*n*=87)* | |
|  | n | *%* | n | *%* | n | *%* | n | *%* |
| Fatigue | 4 | 8.3 | 17 | 17.3 | - | - | 12 | 13.8 |
| Asthenia | 5 | 10.4 | 12 | 12.2 | 5 | 13.2 | 10 | 11.5 |
| Pyrexia | 1 | 2.1 | 9 | 9.2 | 1 | 2.6 | 7 | 8.0 |
| Nasopharyngitis | 3 | 6.3 | 8 | 8.2 | 6 | 15.8 | 6 | 6.9 |
| Back pain | 2 | 4.2 | 8 | 8.2 | 3 | 7.9 | 5 | 5.7 |
| Headache | 1 | 2.1 | 7 | 7.1 | - | - | 6 | 6.9 |
| Exertional dyspnea | 5 | 10.4 | 6 | 6.1 | 1 | 2.6 | 2 | 2.3 |
| Arthralgia | 3 | 6.3 | 6 | 6.1 | 3 | 7.9 | 5 | 5.7 |
| Dizziness | 3 | 6.3 | 5 | 5.1 | 2 | 5.3 | 5 | 5.7 |
| Dyspnea | 2 | 4.2 | 5 | 5.1 | - | - | 5 | 5.7 |
| Myalgia | - | - | 5 | 5.1 | 2 | 5.3 | 3 | 3.4 |
| One patient randomized to placebo received a dose of DAR and so is included in that group. Abbreviations: DAR, darbepoetin alfa. | | | | | | | | |

| **Table S7.** AML cases | | | | | |
| --- | --- | --- | --- | --- | --- |
|  | *24-week double-blind period* | | | *48-week open-label DAR* | |
|  | *Placebo* | *DAR* | |  |  |
| Age, years | 73 | 76 | 78 | 82 | 74 |
| Sex | Male | Female | Male | Male | Male |
| IPSS score/risk at diagnosis | 0.5/int-1 | 1.0/int-1 | 0.5/int-1 | 0.5/int-1 | 1.0/int-1 |
| Baseline WHO 2008 category (locally assessed) | RAEB-1^a^ | RCMD | RARS | RAEB-1 | RAEB-1 |
| Baseline WHO 2008 category (centrally assessed) | RAEB-2 | RAEB-1 | NA^b^ | RAEB-2 | RAEB-1 |
| Baseline marrow blast % | 3.0% | 5.0% | 4.5% | 5.0% | 8.3% |
| IPSS karyotype | Good | Intermediate | Good | Good | Good |
| Trial week progression to AML | 6 | 9 | 21 | 36 | 70 |
| Marrow blasts ≥20% at AML diagnosis | No | Yes | Yes | No | Yes |
| Peripheral blasts ≥20% at AML diagnosis | Yes | Yes | No | Yes | No |
| Status at end of 48-week open-label period | Alive | Alive | Alive | Dead | Alive |
| Abbreviations: AML, acute myeloid leukemia; DAR, darbepoetin alfa; int-1, intermediate-1; IPSS, International Prognostic Scoring System; NA, not available; RAEB, refractory anemia with excess blasts; RARS, refractory anemia with ringed sideroblasts; RCMD, refractory cytopenia with multilineage dysplasia; WHO, World Health Organization.  ^a^Classified as such due to blood myeloblasts of 2%.  ^b^Inadequate sample. | | | | | |

**SUPPLEMENTAL FIGURES**

**Supplemental figure legends**

**Figure S1. Disposition and HI-E rates.** Erythroid response (HI-E) rates are shown alongside study period (ie, 24-week double-blind and 48-week open-label). DAR, darbepoetin alfa; HI-E, hematologic improvement – erythroid response.

**Figure S2. Efficacy by IPSS/IPSS-R risk.** Transfusion rates and erythroid response (HI-E) are shown by IPSS (A, B) and IPSS-R (C, D) risk. DAR, darbepoetin alfa; HI-E, hematologic improvement – erythroid response; int-1, intermediate-1; IPSS, International Prognostic Scoring System; IPSS-R, revised IPSS; IWG, International Working Group; PBO, placebo; RBC, red blood cell.

**Figure S1. Disposition and HI-E rates**

**Figure S2. Efficacy by IPSS/IPSS-R risk status.**

**(A) RBC Transfusions by IPSS**

**(B) HI-E by IPSS status**

**(C) RBC Transfusions by IPSS-R**

**(D) HI-E by IPSS-R status**
